# A Real-World Analysis of the Population with Hepatitis C Virus Infection Affected by Type 2 Diabetes in Italy: Patients’ Characteristics, Comorbidity Profiles and Treatment Patterns

**DOI:** 10.3390/medicina61040614

**Published:** 2025-03-28

**Authors:** Edoardo Giovanni Giannini, Alessandra Mangia, Filomena Morisco, Pierluigi Toniutto, Angelo Avogaro, Stefano Fagiuoli, Claudio Borghi, Francesca Frigerio, Marta Nugnes, Chiara Veronesi, Maria Cappuccilli, Margherita Andretta, Marcello Bacca, Antonella Barbieri, Fausto Bartolini, Gianmarco Chinellato, Andrea Ciaccia, Renato Lombardi, Daniela Mancini, Romina Pagliaro, Loredana Ubertazzo, Luca Degli Esposti, Francesca Romana Ponziani

**Affiliations:** 1 Gastroenterology Unit, Department of Internal Medicine, University of Genoa, 16126 Genoa, Italy; egiannini@unige.it; 2 Liver Unit, Department of Medical Sciences, Fondazione “Casa Sollievo della Sofferenza” IRCCS, 71013 San Giovanni Rotondo, Italy; a.mangia@tin.it; 3 Gastroenterology Unit, Department of Clinical Medicine and Surgery, University of Naples “Federico II”, 80138 Naples, Italy; morisco@unina.it; 4 Hepatology and Liver Transplantation Unit, University of Udine, 33100 Udine, Italy; pierluigi.toniutto@uniud.it; 5 Department of Medicine, Section of Diabetes and Metabolic Diseases, University of Padova, 35128 Padua, Italy; angelo.avogaro@unipd.it; 6 Gastroenterology Hepatology and Transplantation Unit, ASST Papa Giovanni XXIII, 24127 Bergamo, Italy; sfagiuoli@asst-pg23.it; 7 Gastroenterology, Department of Medicine, University of Milan Bicocca, 20126 Milan, Italy; 8 Department of Medical and Surgical Sciences, IRCCS AOU S. Orsola di Bologna, 40138 Bologna, Italy; claudio.borghi@unibo.it; 9 Gilead Sciences, 20124 Milan, Italy; francesca.frigerio@gilead.com; 10 CliCon S.r.l. Società Benefit, Health, Economics & Outcomes Research, 40137 Bologna, Italy; marta.nugnes@clicon.it (M.N.); chiara.veronesi@clicon.it (C.V.); maria.cappuccilli@clicon.it (M.C.); luca.degliesposti@clicon.it (L.D.E.); 11 UOC Assistenza Farmaceutica Territoriale, Azienda ULSS 8 Berica, 36100 Vicenza, Italy; margherita.andretta@aulss8.veneto.it (M.A.); gianmarcochinellato05@gmail.com (G.C.); 12 Programmazione e Controllo di Gestione, ASL Brindisi, 72100 Brindisi, Italy; m.bacca@asl.brindisi.it; 13 SC Farmaceutica Territoriale, Azienda Sanitaria Locale di Vercelli (ASL VC), 13100 Vercelli, Italy; antonella.barbieri@aslvc.piemonte.it; 14 Dipartimento Farmaceutico USL Umbria 2, Coordinatore della Cabina di Regia Regione Umbria Sulla Governance Farmaceutica, 05100 Terni, Italy; fausto.bartolini@uslumbria2.it; 15 Servizio Farmaceutico Territoriale, ASL Foggia, 71100 Foggia, Italy; andrea.ciaccia71@gmail.com (A.C.); renato.lombardi@aslfg.it (R.L.); 16 UOSD Pianificazione Acquisti Farmaci e Beni Sanitari, ASL Brindisi, 72100 Brindisi, Italy; daniela.mancini@asl.brindisi.it; 17 UOC Farmaceutica Territoriale, Azienda Sanitaria Locale Roma 5, 00019 Tivoli, Italy; romina.pagliaro@aslroma5.it; 18 UOC Farmacia Territoriale, ASL Roma 4, 00053 Civitavecchia, Italy; loredana.ubertazzo@aslroma4.it; 19 Liver Unit, CEMAD-Centro Malattie dell’Apparato Digerente, Medicina Interna e Gastroenterologia, Fondazione Policlinico Universitario A. Gemelli IRCCS, 00168 Rome, Italy; 20 Dipartimento di Medicina e Chirurgia Traslazionale, Università Cattolica del Sacro Cuore, 00168 Rome, Italy

**Keywords:** antiviral therapies, direct-acting antivirals, hepatitis C virus, pangenotypic direct-acting antivirals, real-world evidence, type 2 diabetes

## Abstract

*Background and Objectives*: HCV infection represents a main risk factor for type 2 diabetes (T2D). This real-world analysis investigated the HCV-positive (HCV+) population with a T2D co-diagnosis in Italy. *Methods*: From 2017 to 2021, HCV+ patients were identified from administrative databases and stratified into T2D-HCV+ and HCV+-only cohorts in the presence/absence of a T2D diagnosis. Both cohorts were further divided by treatment with direct-acting antivirals (DAAs). The subgroups were compared for demographic variables, comorbidity profiles, most frequent hospitalizations, and drug prescriptions before inclusion. A sensitivity analysis was performed on patients included after 2019, the year of widespread use of pangenotypic DAAs. *Results*: Considering HCV+ patients aged ≥55 years, T2D-HCV+ patients (N = 1277) were significantly (*p* < 0.001) older than HCV+-only (N = 6576) ones and burdened by a worse comorbidity profile (average Charlson index: 1.4 vs. 0.3, *p* < 0.05). Moreover, regardless of T2D presence, DAA-treated patients were older (*p* < 0.001) and had a worse Charlson index than the untreated ones. T2D-HCV+ patients showed tendentially higher hospitalization rates and co-medication prescriptions compared to the HCV+-only patients. After 2019, a trend towards reduced co-medication use in DAA-treated patients was noticed, especially antibiotics and cardiovascular drugs. *Conclusions*: The co-presence of T2D in HCV+ patients resulted in a worse clinical status, as confirmed by the more frequent requirement of hospitalizations and complex polypharmacy regimens.

## 1. Introduction

Hepatitis C virus (HCV) is a hepatotropic RNA virus and the main causative agent of liver diseases, cirrhosis, and hepatocellular carcinoma [1]. It has been reported that between 64 and 103 million people are chronically affected worldwide, making HCV infection a major public health problem [1]. In Western countries, including Italy, the prevalence of HCV infection in the general population ranges from 1% to 2%, with a consistent, declining trend observed in younger cohorts [2,3].

Although much attention from epidemiolocal and medical research has been focused on the effects of HCV infection on liver injury, it should also be underlined that a substantial portion of the clinical burden of HCV is also imputable to other HCV-associated comorbidities, including type 2 diabetes (T2D) and atherosclerosis [4]. The pathogenetic link between HCV and T2D lies in HCV-triggered impairment of the hepatocyte insulin signaling pathway [5]. Evidence has shown that successful interventions on HCV with antiviral therapy infection can lead to glucose metabolism improvement [6,7] and a reduced risk of T2D [8]. Data on the Japanese population revealed a higher prevalence of insulin resistance and T2D in patients with chronic HCV infection compared to HCV-cleared and chronic-HBV groups [9]. A more recent Italian report supported the view that HCV eradication results in reduced T2D rates and better glycemic control [10]. Other international studies have described the prevalence of diabetes among HCV-positive (HCV+) patients between 7.4 and 43.2% [11,12], feasibly explained by the contribution to T2D development of various mechanisms, including direct viral effects, insulin resistance, proinflammatory cytokines, and other immune-mediated phenomena [13]. The close association between HCV and T2D might enhance the direct and indirect damage of HCV to various organs and systems, producing a systemic disease, sometimes independent from the liver injury itself, both in cirrhotic and non-cirrhotic patients [14].

Similar to other countries, in Italy, the prevalence of HCV infection in patients with T2DM is remarkably higher compared to the general population, rising to 9% (range: 4–9%) [15]. An Italian longitudinal cohort study on HCV conducted on the Italian Platform for the Study of Viral Hepatitis Therapies (PITER) confirmed diabetes as one of the most common HCV-related comorbidities, accounting for 14% of cases [16] and reaching a zenith close to 20% among elderly patients (>60 years) [15,17].

Over the last decade, the advent of direct-acting antivirals (DAAs) and then pangenotypic DAAs (pDAAs), used to treat chronic HCV infection across all viral genotypes, has dramatically improved the therapeutic outcomes of these patients, allowing the achievement of a sustained virological response (SVR) in more than 98% of cases, with a good safety profile [18,19]. Nevertheless, the widespread use of DAAs in subjects with HCV infection, together with the complex comorbidity profile of this population and the resultant requirement for polypharmacy regimes, predisposes a significant proportion of patients to an increased risk of drug–drug interactions (DDIs) [20,21]. Real-world studies have shown that up to 70% of HCV-infected patients under pDAA therapy based on glecaprevir–pibrentasvir (GLE-PIB) and sofosbuvir–velpatasvir (SOF-VEL) were taking concomitant medications, and the prevalence of clinically significant DDIs was as high as 40% [22].

The present analysis was conducted in a setting of real clinical practice with the purpose of describing the clinical and pharmacological characteristics of the HCV+ population in Italy, and to assess the prevalence and impact of T2D among these patients. In particular, T2D epidemiology, trends, and the effects of DAAs were investigated by comparing treated and untreated groups. The analysis also examined clinical profiles, comorbidities, and medication use, with a focus on patients aged ≥55 years. A subanalysis of T2D-HCV+ patients from 2019 onward explored treatment trends, aiming to optimize patient management strategies.

## 2. Materials and Methods

### 2.1. Data Source

A retrospective observational analysis was performed using administrative databases including data on healthcare resources/services supplied and reimbursed by the Italian National Healthcare Service (INHS) of a pool of Local Health Units (LHUs) covering approximately 3.5 million health-assisted residents. The following databases were used for this analysis: beneficiaries’ database for patients’ demographics; pharmaceutical database for data on drug prescriptions with their Anatomical Therapeutic Chemical (ATC) code; hospitalization database for the hospital discharge diagnoses classified by the International Classification of Diseases, Ninth Revision, Clinical Modification (ICD-9-CM); exemption database for active payment waiver codes associated with specific disease diagnoses; and outpatient specialist service database for data on specialist visits, diagnostic procedures and laboratory tests.

The dataset used consists solely of anonymized data. Approval has been obtained from the following ethics committees of the involved LHUs: EC authorization “Berica Comitato Etico per le Sperimentazioni Cliniche (CESC) della Provincia di Vicenza”, protocol number 1627, approval date 28 October 2020; EC authorization “Brindisi Comitato Indipendente di Etica Medica”, protocol number 48148, approval date 28 May 2021; EC authorization “Vercelli Comitato Etico Interaziendale A.O. SS. Antonio e Biagio e Cesare Arrigo”, Alessandria, protocol number 0008668, approval date 20 April 2021; EC authorization “Umbria 2 Comitato Etico Regionale Umbria”, protocol number 19414/20/ON, approval date 16 September 2020; EC authorization “Foggia Comitato Etico Interprovinciale Area I”, protocol number 63/CE/20, approval date 3 December 2020; EC authorization “Brindisi Comitato Indipendente di Etica Medica”, protocol number 48148, approval date 28 May 2021; EC authorization “Roma 5 Comitato Etico Lazio 1”, protocol number 1166/CE Lazio 1, approval date 12 October 2020; EC authorization “Roma 4 Comitato Etico Lazio 1”, protocol number 1079/CE Lazio 1, approval date 23 September 2020.

### 2.2. Study Design and Selection Criteria

From January 2017 to December 2021 (inclusion period), all HCV+ patients were identified by a previously described proxy based on the presence of (i) an exemption code specific for HCV (016.070.54); (ii) at least one hospitalization for HCV using the following codes: 070.41 Acute hepatitis C with hepatic coma, 070.44 Chronic hepatitis C with hepatic coma, 070.51 Acute hepatitis C without mention of hepatic coma, 070.54 Chronic hepatitis C without mention of hepatic coma, and 070.7 Unspecified viral hepatitis C or; (iii) at least one prescription of pDAAs (ATC code: J05AP) [23]. Patients with a diagnosis of T2D [identified by at least one discharge diagnosis for T2D (ICD–9–CM code: 250) or exemption code (013) or antidiabetic drugs prescription (ATC code: A10)] [24], during all the available period before HCV diagnosis, were defined as the T2D-HCV+ cohort. Moreover, based on the presence of pDAA prescription during the inclusion period, patients were further stratified into pDAA-treated and untreated. For T2D-HCV+ and HCV+-only patients, the index date was the time of first diagnosis of HCV (exemption or hospitalization), within the inclusion period; for treated patients, the index date was defined as the date of first pDAA prescription. Patients with T2D only were also included for comparative analyses. The characterization period was the whole period of data availability before the index date (at least 12 months), and the follow-up was the whole period of data availability from the index date onwards (at least 12 months). A further analysis was conducted on patients aged ≥55 years, because of the highest susceptibility to T2D among older HCV+ patients.

A sub-analysis was then carried out on T2D-HCV+ patients included from 2019, which is the year of the spread of pDAAs into clinical practice. After that, in July 2018, the WHO issued updated recommendations for the use of pDAA regimens in adults (≥18 years) with chronic HCV infection [25].

### 2.3. Patients’ Baseline Characteristics and Most Frequent Medications Used During Follow-Up

For all patients included, the demographic characteristics were collected at the index date, specifically, age in years; the proportion of patients in the age classes 55–64, 65–74, 75–84, and ≥85 years; and sex distribution expressed as percentage of male subjects. During the characterization period, the general clinical status was investigated using the Charlson comorbidity index [26], which assigns a score to 19 weighted concomitant diseases, searched through drug treatment and hospitalizations in the 12 months preceding inclusion. To better describe the clinical profile of the patients in each cohort, the most common causes of hospitalization and the most frequent drugs prescribed were assessed during the one-year period before inclusion in HCV+ patients with and without diabetes.

### 2.4. Statistical Analysis

Continuous variables are reported as the mean ± standard deviation (SD) and categorical variables as frequencies and percentages. Comparisons between subgroups were conducted using two-way ANOVA for continuous variables and the chi-square test for categorical variables. A *p*-value < 0.05 was considered statistically significant and all the analyses were performed using Stata SE version 17.0 (StataCorp., College Station, TX, USA).

## 3. Results

From a catchment area covering about 3.5 million health-assisted residents, 15,321 (0.4%) patients with a proxy of HCV diagnosis during the inclusion period (2017–2021) were identified: of them, 7803 (10.3%) aged ≥55 years were selected for the analysis (overall HCV+ group). Then, 1227 patients with a diagnosis of T2D, accounting for 15.7% of the included HCV+ patients, were identified as the T2D-HCV+ group. These patients were then compared with HCV+ patients without T2D (HCV+-only group, N = 6576). Moreover, 236,026 T2D patients without HCV infection were included as the control group (T2D-only group).

Table 1 reports the demographic and clinical features of the overall HCV+ population and the subgroups stratified by the presence/absence of HCV infection and by the presence/absence of T2D. T2D-HCV+ patients were significantly older compared to HCV+-only patients (69.8 ± 9.1 vs. 66.9 ± 9.3, *p* < 0.001) but younger than T2D-only patients (72.3 ± 9.6 years, *p* < 0.001). The worst comorbidly profile, assessed through the Charlson index, was observed in the T2D-HCV+ group (1.4 ± 1.2), followed by T2D-only patients (1.3 ± 0.9) and HCV+-only patients (0.3 ± 0.8) (Table 1).

HCV+ patients were then further stratified according to treatment with DAAs. Among the 1227 T2D-HCV+ patients, the untreated numbered 745 (61%) and the DAA-treated numbered 482 (39%). Among 6576 HCV+-only patients, the untreated were 4170 (63%) and the DAA-treated 2406 (37%). As Table 2 shows, in both groups, T2D-HCV+ and HCV+ only, patients who received DAA treatment were significantly older (*p* < 0.001) and had a higher Charlson comorbidity index compared to the untreated ones (*p* < 0.001) (Table 2).

To better describe the comorbidity profile in HCV+ patients with and without T2D, the most common causes of hospitalizations and the most common drug prescriptions were investigated during the 1-year period before inclusion (index date excluded). In the presence of T2D, HCV+ patients tended to have higher rates of hospitalizations due to complications in the hepatobiliary system and pancreas (T2D-HCV+ and HCV+ only: 9.1% and 4.4%), circulatory system (T2D-HCV+ and HCV+ only: 5.9% and 2.4%), digestive system (T2D-HCV+ and HCV+ only: 3.5% and 1.7%), kidney and urinary tract (T2D-HCV+ and HCV+ only: 3.2% and 1.2%), and respiratory system (T2D-HCV+ and HCV+ only: 2.9% and 1.2%). In the T2D-only patients, the most common causes of hospitalization were conditions related to the circulatory system (4.4%), musculoskeletal system and connective tissue (2.4%), respiratory system (1.8%), and digestive system (1.4%).

The detailed pattern of drug prescriptions classified on the second ATC level in T2D-HCV+, HCV+-only, and T2D-only patients in the 1 year prior to the index date is shown in Table 3. In all groups, agents acting on the renin–angiotensin system were the most frequently prescribed treatments and, in general, cardiovascular drugs (antihypertensives, antithrombotic agents, and lipid-modifying agents) were more common in patients with diabetes (with or without HCV infection) compared to HCV+-only patients. More detailed analysis among antithrombotic agents revealed that, in all subgroups, the most commonly prescribed medications were enoxaparin (ATC code B01AB05; 18.6% of T2D-HCV+ patients, 27.0% HCV+-only patients, and 13.1% of T2D patients) and clopidogrel (ATC code B01AC04; 14.4% of T2D-HCV+ patients, 12.6% HCV+-only patients, and 11.8% of T2D patients).

The analysis of drug prescription patterns during the 1-year period before inclusion (index date excluded) was then replicated in T2D-HCV+ and HCV+-only patients, who were stratified by the presence/absence of DAA therapy.

**Figure 1:**
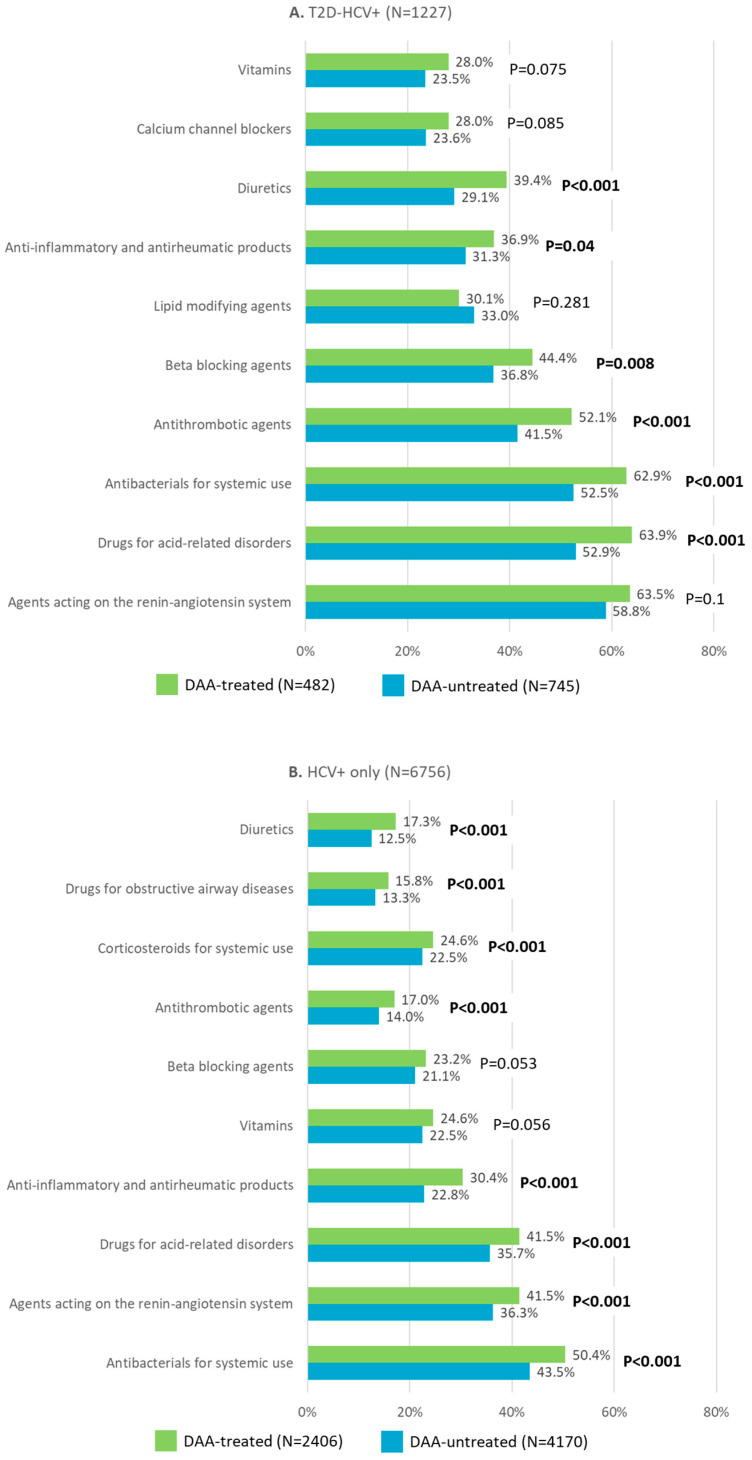
The most frequent treatments during the 1 year before the index date in T2D-HCV+ (**A**) and HCV+-only (**B**) patients stratified by the presence/absence of DAA therapy. Significant *p*-values are highlighted in bold (according to the pairwise comparison by a chi-square test of DAA-treated vs. untreated patients in the T2D-HCV+ group and in the HCV+ group). Abbreviations: DAA, direct-acting antivirals; HCV, hepatitis C virus; T2D, type 2 diabetes.

As shown in Figure 1, the most frequently prescribed drugs in T2D-HCV+ patients were agents acting on the renin–angiotensin system (untreated vs. DAA-treated: 58.8% vs. 63.5%, *p* = 0.100) followed by drugs for acid-related disorders (untreated vs. DAA-treated: 52.9% vs. 63.9%, *p* < 0.001) and antibacterials for systemic use (untreated vs. DAA-treated: 52.5% vs. 62.9%, *p* < 0.001). In HCV+-only patients, stratified by the presence/absence of DAA therapy, the most prescribed drugs were antibacterials for systemic use (untreated vs. DAA-treated: 43.5% vs. 50.4%, *p* < 0.001), followed by agents acting on the renin–angiotensin system (untreated vs. DAA-treated: 36.3% vs. 41.5%, *p* < 0.001) and then drugs for acid-related disorders (untreated vs. DAA-treated: 35.7% vs. 41.5%, *p* < 0.001).

The comparison of the HCV+-only cohort included during the whole inclusion period (2017–2021) with the subset of those who were included after 2019 (the start of the widespread use of pDAAs in clinical practice) indicated a tendential reduction in prescriptions for some co-medications in DAA-treated patients. The proportion of prescriptions for antibacterials for systemic use dropped from 50.4% for the patients included between 2017 and 2021 to 43.3% in those included after 2019, that of agents acting on the renin–angiotensin system dropped from 41.5% to 38.0%, and that of beta-blockers dropped from 23.2% to 20.7% (Table A1 of the Appendix A).

The profiles of drug consumption prescriptions were then analyzed at 1-year follow-up. Considering all treatments, the mean (±SD) number of prescriptions per patient was 9.9 ± 5.9 in the T2D-HCV+ group vs. 5.4 ± 4.8 in the HCV+-only group (*p* < 0.001). The same analysis replicated for chronic treatments alone revealed that the mean (±SD) number of prescriptions per patient was 1.3 ± 1.7 in the T2D-HCV+ group vs. 0.8 ± 1.4 in the HCV+-only group (*p* < 0.001). The focus on the specific co-medications grouped by first-level ATC code in the T2D-HCV+ and HCV+-only cohorts during the first year of follow-up is depicted in Table 4. Anti-hypertensives/cardiac therapies, statins/fibrates, anticoagulants, and drugs for the central nervous system (CNS) were more frequently prescribed in T2D-HCV+ patients than in HCV+-only patients (*p* < 0.001).

## 4. Discussion

HCV infection and diabetes both represent a socio-health emergency, especially because of the close relationship between each other and with cardiovascular disease [13,27]. In this analysis, the comparison between the population with coexisting HCV plus T2D vs. patients with only one of these conditions—HCV or diabetes only—revealed that the presence of both diseases together seems to expose patients to a more complex comorbidity burden requiring polypharmacy regimens. This finding is supported by the Charlson comorbidity index, which was higher in T2D-HCV+ patients compared to T2D-only and HCV+-only patients. Consistent with our findings, a recent review article examining the various non-liver-related complications associated with HCV infection reported that, beyond liver-related issues such as cirrhosis and hepatocellular carcinoma, HCV is linked to several extrahepatic manifestations including diabetes, cardiovascular and cerebrovascular diseases, lymphomas, autoimmune disorders, and mixed cryoglobulinemia. These complications significantly increase the disease’s complexity, contributing to heightened morbidity, mortality, reduced quality of life, and increased healthcare costs [28].

Considering the therapeutic management of HCV, either in the presence or absence of a T2D co-diagnosis, patients who received DAAs were a smaller proportion of the whole HCV+ population, were significantly older, and had a worse clinical status (as documented by the higher Charlson comorbidity index) with respect to the untreated ones. This finding seems to suggest that there is still some room for improvement for greater utilization of DAAs, in line with a recent multinational investigation by Isfordink et al. reporting that, despite unrestricted access to DAAs in high-income countries, several patients remain untreated, likely due to differences in access to care and barriers to treatment [29]. This analysis also showed that considering the entire observation period (2017–2021), HCV+-only patients treated with DAAs had a worse clinical profile compared to their untreated counterparts, as documented by the larger number of concomitant drug prescriptions in the former group. This might be feasibly explained by the different indications initially received from DAAs in the first few years after their introduction, when the prescription was restricted to patients with more advanced liver disease according to the indications of the Italian drug regulatory agency AIFA. Besides, our analysis revealed that this different rate of prescription of some co-medications, especially antibacterials for systemic use, agents acting on the renin–angiotensin system, and beta-blockers, between DAA-treated and untreated HCV+ patients disappeared in those who also had T2D. Taken together, these findings seem to suggest that HCV+ patients treated with DAAs had more advanced liver disease, but since the difference in terms of co-treatment patterns faded in the presence of T2D, it can be hypothesized that T2D represents the main culprit in complicating the comorbidity profile in HCV+ patients, rather than HCV infection by itself. This view is consistent with a study by Angel et al. that suggested that the co-presence of diabetes may increase clinical complexity and the burden of comorbidities in HCV patients, potentially affecting treatment decisions and outcomes [30].

The profiles of drug prescriptions at 1-year follow-up revealed that the number of prescriptions per patient was almost double in T2D-HCV+ patients compared to HCV+-only patients. Unsurprisingly, when detailing the type of concomitant medications, cardiovascular drugs were more common in T2D patients (with or without HCV infection) compared to HCV+-only patients. The view that T2D can be one main driver of the clinical worsening of HCV+ patients is supported by our data on hospitalizations, as patients with concomitant HCV infection and T2D displayed higher rates of hospitalizations, especially those related to the hepatobiliary system and pancreas. In particular, anti-hypertensives/cardiac therapies, statins/fibrates, anticoagulants, and drugs for the CNS were more frequently prescribed in T2D-HCV+ patients than in HCV+-only patients. It is well known that with the aging population, many patients are treated with several concomitant medications for chronic diseases, especially lipid-lowering agents, and antihypertensive drugs, which makes them highly susceptible to potential DDIs [31]. In consideration of the potential DDI risk, the European Association for the Study of the Liver (EASL) recommends an in-depth examination of patients’ history for all prescribed medications, including over-the-counter drugs and herbal and vitamin preparations, and any abused drugs before starting DAAs [19].

A recent Delphi consensus project gathered a panel of Italian cardiologists and hepatologists to evaluate DDIs in HCV-infected patients on DAA therapy in the presence of comorbidities and cardiovascular risk. The experts discouraged the choice of dose changes, drug substitution, and discontinuation of concomitant cardiovascular drugs, in view of the complex management of cardiovascular issues and the elevated risks associated with therapy changes [20]. Thus, the main efforts should, rather, be targeted at a careful evaluation of potential DDIs associated with each oral DAA, and hepatologists should opt for the antiviral DAA combination presenting the lowest likelihood of potential interactions [20].

Besides the advantages of using data obtained from real-world clinical settings on large, unselected populations, this analysis has some limitations to be acknowledged. Administrative databases are meant to trace drugs and healthcare services covered by the National Health System and are not for research purposes. Thus, information on the clinical picture and concomitant disease severity might be incomplete or lacking. For the same reason, some important variables, like viremia, body mass index, blood glucose, and glycated hemoglobin, could not be traced within the databases. Moreover, the patients with HCV were identified using specific codes (i.e., exemption code specific for HCV, hospitalization for HCV, and prescription of pDAAs), so patients with HCV infection but without a specific code (e.g., those with HCV-related cirrhosis having an exemption code for cirrhosis only) were missed. However, in spite of these flaws and the lack of serologic data, previous evidence has supported the good predictive value of the presence of an HCV code for the diagnosis of HCV infection [32]. Besides, the possible correlation between DAA treatment and improved T2D control could not be evaluated because the databases used did not allow us to extrapolate laboratory data on the indexes of T2D status (fasting blog glucose, HbA1c, and insulin). Another potential fault might be an underestimation of the Charlson index since the diagnoses of the 19 concomitant diseases were proxied using hospitalization codes or ATC codes for medications; hence, comorbidities without treatment or hospitalization were not weighted. Moreover, data on prescriptions were extracted from pharmaceutical databases; thus, the use of OTC products and vitamin supplementation could not be retrieved. Lastly, the study included patients between 2017 and 2021, but the potential impact of the COVID-19 pandemic on T2D and HCV-related outcomes was not specifically assessed. Given the significant healthcare disruptions and changes in drug prescription behaviors associated with COVID-19 infection and lockdowns, this may have influenced some of the findings, particularly those from 2020 to 2021 [33,34]. Future studies should account for pandemic-related variables to better isolate the effects of clinical and demographic factors.

## 5. Conclusions

The study showed that T2D-HCV+ patients were characterized by elevated comorbidity and polypharmacy regimens compared to HCV+-only and T2D-only patients. Given the known interconnected pathogenic mechanisms among HCV infection, diabetes, and cardiovascular disease, it is not surprising that the majority of prescribed medications were cardio-metabolic agents. These findings underscore the significant concern regarding potential interactions between antiviral therapy and pre-existing chronic treatments. Considering the general medical consensus against altering dosage, substituting drugs, or discontinuing cardiovascular medications, a meticulous assessment of potential DDIs is strongly recommended before initiating antiviral therapy. The data emerging from the present analysis could provide a rationale for further studies aimed at optimizing the therapeutic management of HCV+ patients affected by T2D or other concomitant cardiovascular risk factors.

## Figures and Tables

**Table 1:** Baseline characteristics at the index date during the inclusion period of the overall HCV+ population, T2D-HCV+, HCV+-only, and T2D-only patients. Significant *p*-values are highlighted in bold (according to the chi-square test used to compare T2D-HCV+ vs. HCV+ only vs. T2D only).

	Overall HCV+ (N = 7803)	T2D-HCV+ (N = 1227)	HCV+ Only (N = 6576)	T2D Only (N = 236,026)	*p*-Value
Age at index date, years, mean (±SD)	67.3 (±9.3)	69.8 (±9.1)	66.9 (±9.3)	72.3 (±9.6)	**<0.001**
Age classes					
55–64 years, N (%)	3606 (46.2%)	412 (33.6%)	3194 (48.6%)	56,195 (23.8%)	
65–74 years, N (%)	2126 (27.2%)	378 (30.8%)	1748 (26.6%)	81,878 (34.7%)	
75–84 years, N (%)	1789 (22.9%)	382 (31.1%)	1407 (21.4%)	70,564 (29.9%)	
≥85 years, N (%)	282 (3.6%)	55 (4.5%)	227 (3.5%)	27,389 (11.6%)	
Male gender, N (%)	3966 (50.8%)	716 (58.4%)	3250 (49.4%)	122,624 (52.0%)	**<0.001**
Charlson comorbidity index, mean (±SD)	0.5 (±1.0)	1.4 (±1.2)	0.3 (±0.8)	1.3 (±0.9)	**<0.05**
0, N (%)	5315 (68.1%)	149 (12.1%)	5166 (78.6%)	12,612 (5.3%)	
1, N (%)	1685 (21.6%)	732 (59.7%)	953 (14.5%)	160,882 (68.2%)	
≥2, N (%)	803 (10.3%)	346 (28.2%)	457 (6.9%)	62,532 (26.5%)	

Abbreviations: HCV, hepatitis C virus; SD, standard deviation; T2D, type 2 diabetes.

**Table 2:** Baseline characteristics of the T2D-HCV+ and HCV+-only patients stratified by the presence/absence of DAA therapy. Significant *p*-values are highlighted in bold (according to the chi-square test used to compare patients with DAA-treated vs. -untreated T2D-HCV+ and patients with DAA-treated vs. -untreated HCV+ only).

	T2D-HCV+ (N = 1227)			HCV+ Only (N = 6576)		
	Untreated (N = 745)	DAA-Treated (N = 482)	*p*-Value	Untreated (N = 4170)	DAA-Treated (N = 2406)	*p*-Value
Age at index date, years, mean (±SD)	69.1 (±8.85)	70.9 (±9.39)	**<0.001**	66.4 (±9.00)	67.65 (±9.66)	**<0.001**
Age classes						
55–64 years, N (%)	269 (36.11%)	143 (29.67%)		2078 (49.83%)	1116 (46.38%)	
65–74 years, N (%)	246 (33.02%)	132 (27.39%)		1159 (27.79%)	589 (24.48%)	
75–84 years, N (%)	201 (26.98%)	181 (37.55%)		806 (19.33%)	601 (24.98%)	
≥85 years, N (%)	29 (3.89%)	26 (5.39%)		127 (3.05%)	100 (4.16%)	
Male gender, N (%)	444 (59.60%)	272 (56.43%)	0.272	2082 (49.93%)	1168 (48.55%)	0.280
Charlson comorbidity index, mean (±SD)	1.29 (±1.10)	1.57 (±1.31)	**<0.001**	0.29 (±0.73)	0.45 (±1.01)	**<0.001**
0, N (%)	104 (13.96%)	45 (9.34%)		3364 (80.67%)	1802 (74.90%)	
1, N (%)	459 (61.61%)	273 (56.64%)		577 (13.84%)	376 (15.63%)	
≥2, N (%)	182 (24.43%)	164 (34.02%)		229 (5.49%)	228 (9.48%)	

Abbreviations: DAA, direct-acting antivirals; HCV, hepatitis C virus; SD, standard deviation; T2D, type 2 diabetes.

**Table 3:** The most frequent treatments classified on the second ATC level, during the 1 year prior to the index date, in T2D-HCV+, HCV+-only, and T2D-only patients. Significant *p*-values are highlighted in bold (according to the chi-square test used to compare T2D-HCV+ vs. HCV+ only vs. T2D only).

ATC Code (2nd Level)	Description	T2D-HCV+ (N = 1227)	HCV+ Only (N = 6576)	T2D Only (N = 236,026)	*p*-Value
C09	Agents acting on renin–angiotensin system	744 (60.6%)	2514 (38.2%)	156,387 (66.3%)	**<0.001**
A02	Drugs for acid-related disorders	702 (57.2%)	2487 (37.8%)	121,345 (51.4%)	**<0.001**
J01	Antibacterials for systemic use	694 (56.6%)	3029 (46.1%)	129,100 (54.7%)	**<0.001**
B01	Antithrombotic agents	560 (45.6%)	1462 (22.2%)	127,375 (54.0%)	**<0.001**
C07	Beta-blocking agents	488 (39.8%)	1437 (21.9%)	84,981 (36.0%)	**<0.001**
M01	Anti-inflammatory and antirheumatic products	411 (33.5%)	1681 (25.6%)	92,791 (39.3%)	**<0.001**
C03	Diuretics	407 (33.2%)	939 (14.3%)	66,439 (28.1%)	**<0.001**
C10	Lipid-modifying agents	391 (31.9%)	740 (11.3%)	124,353 (52.7%)	**<0.001**
C08	Calcium channel blockers	311 (25.3%)	845 (12.8%)	56,264 (23.8%)	**<0.001**
L01	Antineoplastic agents	19 (1.5%)	75 (1.1%)	2615 (0.1%)	0.331

Abbreviations: ATC, Anatomical Therapeutic Chemical; HCV, hepatitis C virus; T2D, type 2 diabetes.

**Table 4:** Top three most frequent co-medications prescribed during the first year of follow-up, grouped by first-level ATC code in the T2D-HCV+ and HCV+-only cohorts. Significant *p*-values are highlighted in bold (according to the chi-square test used to compare T2D-HCV+ vs. HCV+ only).

	Overall HCV+ (N = 7803)	T2D-HCV+ (N = 1227)	HCV+ Only (N = 6576)	*p*-Value
C—Anti-hypertensives/cardiac therapy, N (%)	213 (2.7%)	55 (4.5%)	158 (2.4%)	**<0.001**
Enalapril	109 (1.4%)	19 (1.5%)	90 (1.4%)	
Verapamil	52 (0.7%)	14 (1.1%)	38 (0.6%)	
Ranolazine	52 (0.7%)	21 (1.7%)	31 (0.5%)	
C—Statins/Fibrates, N (%)	963 (12.3%)	305 (24.9%)	658 (10.0%)	**<0.001**
Atorvastatin	591 (7.6%)	186 (15.2%)	405 (6.2%)	
Simvastatin	217 (2.8%)	79 (6.4%)	138 (2.1%)	
Rosuvastatin	110 (1.4%)	31 (2.5%)	79 (1.2%)	
B—Anticoagulants, N (%)	428 (5.5%)	97 (7.9%)	331 (5.0%)	**<0.001**
Warfarin	209 (2.7%)	49 (4.0%)	160 (2.4%)	
Rivaroxaban	104 (1.3%)	19 (1.5%)	85 (1.3%)	
Apixaban	71 (0.9%)	15 (1.2%)	56 (0.9%)	
N—CNS drugs, N (%)	212 (2.7%)	56 (4.6%)	156 (2.4%)	**<0.001**
Quetiapine	75 (1.0%)	17 (1.4%)	58 (0.9%)	
Fentanyl	76 (1.0%)	27 (2.2%)	49 (0.7%)	
Risperidone	22 (0.3%)	6 (0.5%)	16 (0.2%)	

Abbreviations: ATC, Anatomical Therapeutic Chemical; CNS, central nervous system; HCV, hepatitis C virus; T2D, type 2 diabetes. Notes: Anti-hypertensive/cardiac therapy: aliskiren (ATC code C09XA02), bosentan (ATC code C02KX01), sartans (ATC code C09C), enalapril (ATC code C09AA02), prazosin (ATC code C02CA01), ranolazine (ATC code C01EB18), verapamil (ATC code C08DA01). Statins/fibrates: atorvastatin (ATC code C10AA05, C10BA05), lovastatin (ATC code C10BA01, C10AA02), simvastatin (ATC code C10AA01), pitavastatin (ATC code C10AA08), pravastatin (ATC code C10AA03), rosuvastatin (ATC code C10AA07), gemfibrozil (ATC code C10AB04). Anti-coagulants: warfarin (ATC code B01AA03), rivaroxaban (ATC code B01AF01), apixaban (ATC code B01AF02), edoxaban (ATC code B01AF03), dabigatran (ATC code B01AE07). CNS drugs: (al)fentanyl (ATC code N02AB03), (hydr)oxycodone (ATC code N02AA05), quetiapine (ATC code N05AH04), clozapine (ATC code N05AH02), paliperidone (ATC code N05AX13), oxcarbazepine (ATC code N03AF02), aripiprazole (ATC code N05AX12), risperidone (ATC code N05AX08).

## Data Availability

All data used for the current study are available in an aggregated form upon reasonable request to CliCon S.r.l. Società Benefit, which is the body entitled to data treatment and analysis by Local Health Units.

## Appendix A

**Table A1:** Most common treatments prescribed at the second ATC level within the 1 year preceding the index date for patients diagnosed with HCV only, categorized by the presence or absence of DAA therapy, and for patients included from 2019.

		HCV+ Only (N = 1289), Included from 2019	
ATC Code (2nd Level)	Description	Untreated (N = 4170)	DAA-Treated (N = 2406)
J01	Antibacterials for systemic use	185 (42.6%)	370 (43.3%)
C09	Agents acting on the renin–angiotensin system	178 (41.0%)	325 (38.0%)
A02	Drugs for acid-related disorders	163 (37.6%)	325 (38.0%)
M01	Anti-inflammatory and antirheumatic products	116 (26.7%)	225 (26.3%)
A11	Vitamins	91 (21.0%)	189 (22.1%)
C07	Beta-blocking agents	88 (20.3%)	234 (27.4%)
B01	Antithrombotic agents	88 (20.3%)	177 (20.7%)
H02	Corticosteroids for systemic use	77 (17.7%)	142 (16.6%)
R03	Drugs for obstructive airway diseases	57 (13.1%)	*
C03	Diuretics	56 (12.9%)	153 (17.9%)

* not present in the top most frequently prescribed drugs list.
